# Atraumatic Displaced Femoral Neck Fracture Postpartum: A Case Report and Review of the Literature

**DOI:** 10.5435/JAAOSGlobal-D-19-00037

**Published:** 2019-09-17

**Authors:** Samantha Tayne, David Fralinger, Arif Ali

**Affiliations:** From the Department of Orthopedic Surgery, University of Illinois at Chicago, Chicago, IL (Dr. Tayne, Dr. Fralinger, and Dr. Ali), and the Orthopaedic Surgery Specialists, LTD, Park Ridge, IL (Dr. Ali).

## Abstract

Hip pathology during pregnancy may include transient osteoporosis of the hip or osteonecrosis associated with pregnancy. Rarely, hip pathology during pregnancy may result in a fragility fracture or advanced collapse of the femoral head, necessitating surgical treatment. We present a case of a 32-year-old woman who postpartum was found to have a displaced right femoral neck fracture and an area of focal edema in the left femoral head with mild flattening of the articular surface. She was successfully treated with a total hip arthroplasty on the right, and a follow-up MRI of the left hip showed near-complete resolution of the edema in the femoral head. This case underlines the importance of maintaining a clinical suspicion for pathology of the hip during pregnancy and the subsequent consequences of a missed diagnosis.

Pelvic or hip pain is a common report during pregnancy; however, rare instances of more serious hip pathology exist that could lead to fragility fractures, including transient osteoporosis of the hip (TOH) and osteonecrosis (ON).^1,2^ Determining which patients have pain that is benign and which complaints require additional workup can be difficult. In pregnancy, transient osteoporosis can occur even in otherwise healthy individuals, with bony edema and demineralization, leading to a potential for fracture without notable trauma.^1–3^ The etiology of TOH is unknown, but it does typically resolve over a period of 4 to 9 months.^3^ ON of the femoral head during pregnancy is a separate pathology and again may occur in healthy individuals who otherwise have no known risk factors for ON. The etiology is again not completely understood, but ON is more likely to progress beyond pregnancy and the postpartum period. Both transient osteoporosis and ON that occur during pregnancy lead to long-term consequences when unrecognized.^2,4–6^

Pathology of the hip during pregnancy or postpartum is often identified late, necessitating a total hip arthroplasty over internal fixation in an age group where preservation of the native anatomy is preferred.^7,8,9,10,11,12,13,14,15^ There is lack of information in the orthopaedic literature regarding diagnosis and treatment of hip pathology during pregnancy, and therefore lack of recognition on the part of orthopaedic surgeons. We present a young female patient who was misdiagnosed with radicular pain during her third trimester and who went on to develop a femoral neck fracture on the right with underlying bone marrow edema and an area of focal edema in the left femoral head, both identified postpartum.

The patient was informed that information regarding her case would be submitted for publication, and the patient provided her consent.

## Case Report

A 32-year-old Caucasian woman presented to an outside orthopaedic spine surgeon at 31 weeks of pregnancy because of a right leg pain and difficulty walking. The patient reported that the pain had come on gradually and it had caused her to go from walking independently to requiring the use of a cane and to eventually a walker. The patient underwent an MRI of the lumbar spine 1 month prior to delivery because of concerns that her pain and weakness were radicular in nature. A limited MRI of the lumbar spine, with only sagittal and axial reconstruction and without extension to the pelvis or hips, showed a mild disk bulge at L3-4 and L4-5. She was prescribed a Medrol Dosepak for a herniated disk and right lower extremity radiculopathy.

**Figure 1:**
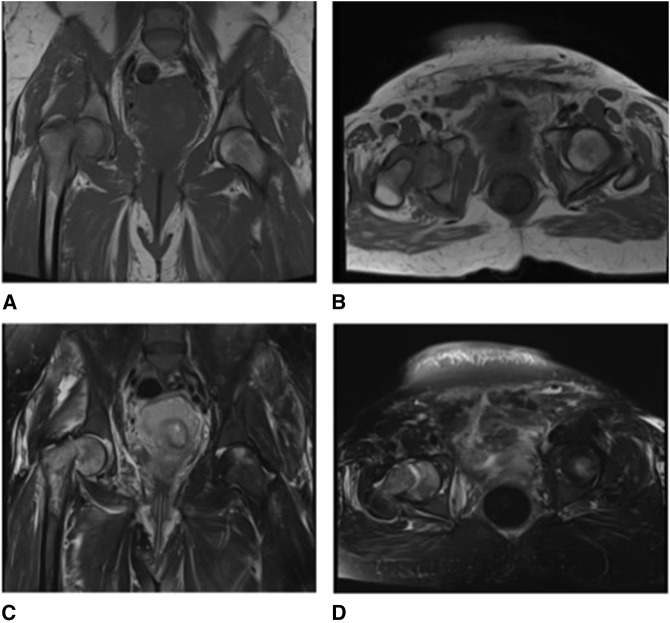
T1 coronal view (**A**), T1 axial view (**B**), T2 coronal view (**C**), and T2 axial view (**D**) of pelvic MRI showing a displaced right femoral neck fracture and evidence of early osteonecrosis in the left femoral head.

**Figure 2:**
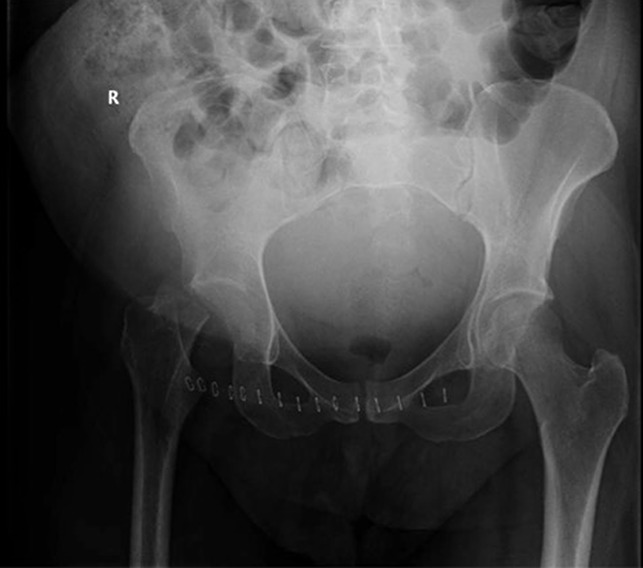
AP pelvis showing a displaced right femoral neck fracture.

The patient continued to experience pain and difficulty ambulating. She presented to the obstetric service at 38 weeks with elevated blood pressure and headaches, and was admitted to labor and delivery for induction of labor. After 16 hours of labor, the decision was made to perform a cesarean section because of concerns for worsening of the right lower extremity radiculopathy and suspected fetal macrosomia, complicated by intrapartum hemorrhage. After her cesarean and delivery, the patient experienced increased severe pain in her right hip and was unable to ambulate. The obstetrics and gynecology team first consulted the neurology service, and an MRI of the pelvis was recommended to evaluate for possible compressive femoral nerve neuropathy. The neurology team also started her on a low dose of prednisone. On the pelvic MRI, she was found to have a displaced right femoral neck fracture with signs of femoral head bone marrow edema and a focal area of bone edema in the left femoral head with mild flattening of the femoral head (Figure 1). The radiologist described the areas of edema in both hips as possible ON. At this point, the orthopaedic service was consulted and a pelvic radiograph was obtained (Figure 2).

**Figure 3:**
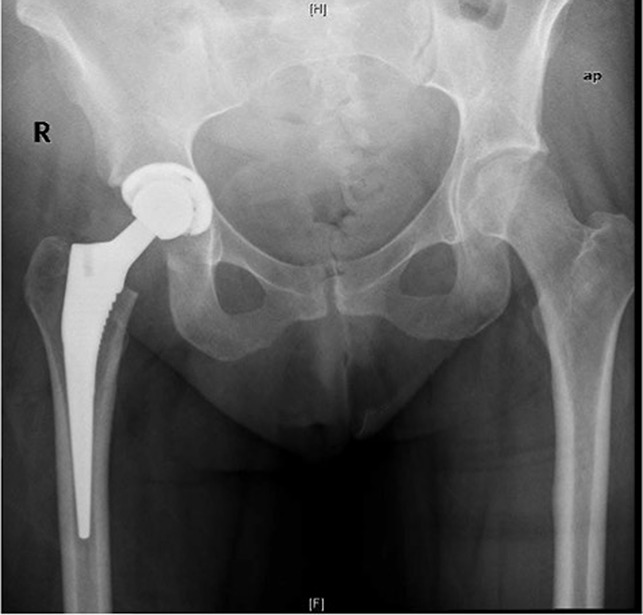
AP pelvis after right total hip arthroplasty.

The patient had no known history of trauma or falls during her pregnancy. She further had no known history of rheumatologic disease nor oncologic disease, prolonged steroid use, smoking, or alcohol abuse. The patient did subsequently undergo an oncologic and rheumatologic workup, including antinuclear antibody, rheumatoid factor, and protein electrophoresis, all of which were found to be negative. We believe that the patient had an undiagnosed nondisplaced fracture that went on to displace during her cesarean, most likely as a result of previously undiagnosed hip pathology associated with pregnancy. Since the timing of her fracture was unclear and because of the degree of displacement, we discussed with the patient that open reduction and internal fixation had a high risk of failure and decided to proceed with arthroplasty. Five days postpartum, the patient underwent a right total hip arthroplasty (Figure 3). The right femoral head and neck were sent for pathology, which reported a “thin trabecular bone structure and recent medullary hemorrhage, consistent with fracture site.” No malignant pathologic process, necrosis, or any other significant pathology was noted. Although the radiologist in his report associated the bony edema in the right hip with ON underlying the femoral neck fracture, the diffuse area of edema seen on the MRI and noted in the pathology report, without mention of bony necrosis, were more consistent with TOH.

Beginning on postoperative day one, the patient was allowed to weight bear as tolerated and began to work with physical therapy. The patient did not report any pain in the left hip or groin; therefore, despite an MRI showing a small focal area of edema, she was allowed to weight bear bilaterally. She was found to have hypoalbuminemia (albumin level 1.6) attributed to malnutrition, for which she was referred to a nutritionist as an outpatient.

**Figure 4:**
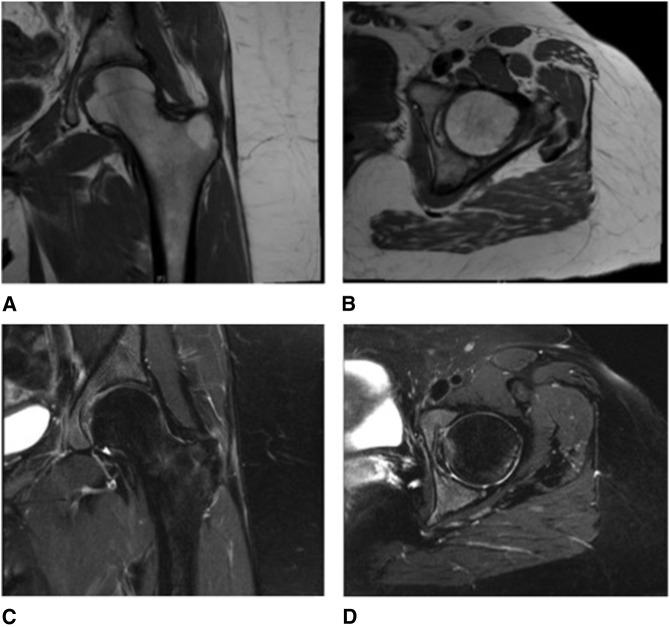
T1 coronal view (**A**), T1 axial view (**B**), T2 coronal view (**C**), and T2 axial view (**D**) of left hip MRI performed 6 months postpartum, showing near complete resolution of osteonecrosis of left femoral head.

At 6 months postpartum, the patient was ambulating with a nonantalgic gait and had full and painless range of motion of bilateral hips. The patient also underwent a repeat MRI of the left hip to monitor the area of edema in the left femoral head (Figure 4). This MRI showed near-complete resolution of the previous findings of edema and flattening of the femoral head on the left, again more consistent with a diagnosis of TOH.

## Discussion

Rare subgroups of women suffer from TOH or ON associated with pregnancy, typically occurring in the third trimester or in the immediate postpartum period, resulting in bone marrow edema and possible fracture.^1–3,15^ MRI is the best method to detect early signs of TOH and ON; however, these diagnoses can be very difficult to differentiate radiographically or clinically. On MRI, TOH shows up as a low signal intensity on T1-weighted images and high signal intensity on T2-weighted images, with a pattern of diffuse edema that may extend into the femoral neck and into the intertrochanteric region.^1,6,9^ ON may be more focal or well-demarcated with the decreased signal intensity of T1- and T2-weighted images and often a double-density signal around the rim.^6^

Importantly, TOH will typically resolve with conservative treatment, whereas ON will continue to advance.^1,6^ The etiology of TOH is unknown, but TOH appears to occur in three stages. In the first stage, acute pain is caused by edema within the bone; the second stage involves resorption and demineralization of the bone, whereas the last stage typically demonstrates clinical and radiographic resolution.^1^ Some authors suggest a possible association between TOH and ON, indicating that TOH may infrequently result in bone marrow edema severe enough to cause vascular disruption, leading to ON.^1,3^ The etiology of ON remains incompletely understood, as well, and may relate to hormonal changes, venous stasis possibly from venous compression of the fetus, or administration by corticosteroids during pregnancy.^2,4–6^ We do not know a true incidence of TOH or ON during pregnancy because many of the women with more mild cases are likely never identified. However, the consequences of late diagnosis may be severe as one study reported a fracture rate of 18.2% in those patients identified with TOH.^15^

Our patient's presentation is similar to that in previous reports in which the diagnosis of TOH is not identified until a fracture has already occurred through the weakened bone, necessitating surgical treatment.^7,8,9,10,11,12,13,14^ In other cases, the diagnosis of ON is also delayed, requiring more extensive surgical treatment.^2,4,5^ On first presentation, many patients are treated conservatively, without imaging, for pain presumed to be related to pregnancy, hip dysplasia, or referred low back pain and remain undiagnosed until they present with difficulty in walking.^7,8,9,10,11,12,13,14^ In addition, complicating the diagnosis is often limited diagnostic imaging because of the understandable reluctance to expose pregnant women to radiation.

Three case reports, all describing TOH with bilateral femoral neck fractures during pregnancy, also describe a metabolic or nutritional disorder, including hypophosphatemic rickets, vitamin D deficiency, and anorexia nervosa.^8,11,12^ A case-control study evaluating risk factors for developing TOH during pregnancy found a notable increase in TOH in women who suffered from dental issues and in patients who performed little or no exercise during childhood.^15^ These reports seem to indicate an increased risk of TOH and femoral neck fractures with already known metabolic bone disease or nutritional deficits. Our patient was found to have hypoalbuminemia, also suggestive of nutritional deficiency.

Another report described a patient who had received two intramuscular injections of betamethasone for fetal lung maturity because of early delivery for eclampsia. This patient had reported hip pain at the end of her pregnancy and may have had early signs of ON, which may have been further accelerated with the steroid injections.^4^ This does not suggest that imperative treatment for the baby should not be used because of the potential for hip pathology, but rather both the obstetrician and the orthopaedic surgeon should be aware of the possible diagnosis of ON and TOH in the mother and manage it accordingly, especially if steroid treatment is used.

Once identified, treatment or activity modification to prevent progression of TOH to fracture or the progression of ON to advanced collapse is poorly defined. Treatment with diphosphonate therapy, calcitonin, or teriparatide is reported in small case series to shorten recovery time for TOH; however, there are significant risks to the fetus associated with diphosphonate exposure.^1,5^ More likely, patients during pregnancy should be treated with activity modification and protected, partial weight-bearing, due to the increased risk of fracture from bone demineralization and with calcium and vitamin D supplementation.^3^ Further medical treatment with diphosphonates may be initiated postpartum when safer for the baby. Postpartum patients may even be cautioned that breastfeeding may further promote TOH because prolactin reduces estradiol and progesterone through the hypothalamic-pituitary-ovarian axis.^1,5^ For those patients who have sustained notable ON with collapse or fracture, surgical treatment with percutaneous pinning, open reduction and internal fixation, use of free vascularized fibular graft, and total hip arthroplasty have all been described and should be utilized at the comfort level and discretion of the treating surgeon.^4,5,6,7,8,9,10,11,12,13,14,15^

Owing to the severe consequences of missed or misdiagnosis, awareness and further understanding of TOH and ON during pregnancy is needed in both obstetric and orthopaedic practices. Understanding the risk factors associated with TOH and ON during pregnancy may help determine when further imaging is necessary to facilitate diagnosis, specifically MRI, which is the safest modality during the third trimester and the most reliable modality to detect early signs of both pathologies. Follow-up imaging is also needed to visualize resolution of TOH or to monitor the progression of ON and to aid in activity restrictions. Further studies on medical treatment and activity modification to prevent femoral neck fracture or advanced collapse of the femoral head is prudent.
